# A Comparative Study of Nafion 212 and Sulfonated Poly(Ether Ether Ketone) Membranes with Different Degrees of Sulfonation on the Performance of Iron-Chromium Redox Flow Battery

**DOI:** 10.3390/membranes13100820

**Published:** 2023-09-30

**Authors:** Enrui Bai, Haotian Zhu, Chuanyu Sun, Guanchen Liu, Xiaoyin Xie, Chongyang Xu, Sheng Wu

**Affiliations:** 1 Yantai Research Institute, Harbin Engineering University, Yantai 264003, China; s321517001@163.com (E.B.); zht18732497702@163.com (H.Z.); 2 School of Chemistry and Chemical Technology, Hubei Polytechnic University, Huangshi 435003, China; 3 School of Electrical Engineering and Automation, Harbin Institute of Technology, Harbin 150001, China; chuanyu.sun@hit.edu.cn; 4 Hubei Xinye Energy-Storage Co., Ltd., Huangshi 435100, China; gcliu@hbpu.edu.cn

**Keywords:** iron-chromium redox flow battery, SPEEK, degrees of sulfonation, physicochemical performance, single-cell efficiency

## Abstract

For an iron-chromium redox flow battery (ICRFB), sulfonated poly(ether ether ketone) (SPEEK) membranes with five various degrees of sulfonation (DSs) are studied. To select the SPEEK membrane with the ideal DS for ICRFB applications, the physicochemical characteristics and single-cell performance are taken into consideration. Following all the investigations, it has been determined that the SPEEK membrane, which has a DS of 57% and a thin thickness of 25 μm, is the best option for replacing commercial Nafion 212 in ICRFB. Firstly, it exhibits a better cell performance according to energy efficiency (EE) and coulombic efficiency (CE) at the current density range between 40 mA cm^−2^ and 80 mA cm^−2^. Additionally, it has a more stable EE (79.25–81.64%) and lower discharge capacity decay rate (50%) than the Nafion 212 (EE: 76.74–81.45%, discharge capacity decay: 76%) after 50 charge–discharge cycles, which proves its better oxidation stability as well. In addition, the longer self-discharge time during the open-circuit voltage test further demonstrates that this SPEEK membrane could be employed for large-scale ICRFB applications.

## 1. Introduction

The redox flow battery (RFB) [1] was proposed by NASA in 1974, which possesses the superiority of environmentally friendly, high safety, recyclable electrolyte, long cycle life, and cost-effective life cycle [2,3]. After 50 years of investigations and developments, it has evolved into one of the best methods for a large-scale energy storage system to solve the problems of intermittent renewable energies, such as wind and solar [4]. Among them, the all-vanadium redox flow battery (VRFB) has been implemented in a series of relatively mature demonstration projects due to its stable performance [5], which proves the practicability of RFB based on long-term and large-scale energy storage at the grid level [6]. However, the high cost of vanadium greatly restricts the further development and wide industrial application of the VRFB [7,8]. Nowadays, with the optimization of electrolyte and hydrogen evolution problems [9], ICRFB with substantial and economical materials has come back into view again [10].

**Figure 1:**
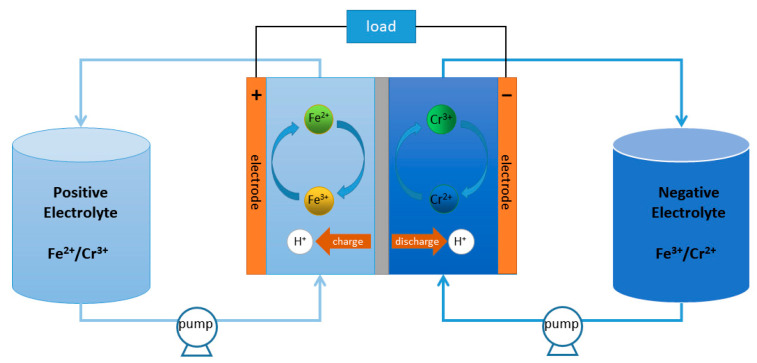
The structure of ICRFB.

It is clearly shown in Figure 1 that the ICRFB is primarily made up of the membrane, electrolytes, electrodes, and other components. The membrane controls the passage of protons or metal ions in the microstructure and divides the electrolytes at the two poles, ensures the balance of positive and negative electrode charges, and forms a closed circuit of the battery [11]. In the ICRFB system, due to the different concentrations of Fe/Cr ions on both sides, the metal ions will continue to migrate to the other side through the membrane with the change in time under the influence of osmotic pressure [12]; this crossover effects will reduce the capacity and efficiencies of it [13]. Therefore, it is very important to prepare membranes with a low permeability and a high selectivity of active substances to improve the efficiencies of ICRFB [14].

Nafion, a perfluorinated membrane from Chemours (Wilmington, DE, USA) was utilized extensively due to its excellent electrical conductivity and good durability that lasts for years [15,16]. However, the poor ion selectivity and extremely high cost (from USD 500 to USD 1000 per square meter) [17] hinder its further application for large-scale industries. To minimize the cost of the RFB, a number of non-fluorinated sulfonated aromatic membranes have been studied by researchers, including sulfonated poly(fluorenyl ether ketone) (SPFEK) [18], sulfonated polyimide (SPI) [19], sulfonated poly(phthalazinone ether ketone) (SPPEK) [20], sulfonated poly(arylene ether sulfone ketone) (SPESK) [21], sulfonated poly(ether ether ketone) (SPEEK) [22], and so on. SPEEK is the most attractive among them, and due to its advantages of low cost, excellent performance, and ease of preparation, it possesses a huge potential to be employed for large-scale applications [23].

In past works, Xi et al. [24] have studied the characteristics of SPEEK membranes with various degrees of sulfonation (DSs) between 57% and 87% for VRFB. However, considering the special operating temperature (65 °C for ICRFB [25], while room temperature for VRFB [26]) and different electrolyte systems, the excessive DS may reduce the properties of SPEEK, especially on ion selectivity, mechanical properties, and chemical stability. In this work, five different SPEEK membranes with DSs between 43% and 62% were prepared. The tests of physicochemical properties and ion selectivity were used to determine the best DS for SPEEK membranes in ICRFB applications, and the real performance of these membranes assembled in the ICRFBs has been investigated by the single-cell tests. Additionally, thickness is one of the crucial factors of the membrane resistance, so it should be considered to reduce the thickness to improve the single-cell performance of the membranes. In addition, three Nafion membranes with various thicknesses have been studied and compared for performance in the ICRFB in our past work [27], and Nafion 212 has been proven to be the best of them. Therefore, this research selected Nafion 212 as the reference for comparison, in order to seek a better membrane used in the ICRFB.

## 2. Materials and Methods

### 2.1. Materials

Poly(ether ether ketone) (PEEK 450PF) was provided from Victrex, London, UK. Nafion 212 membranes were provided from Chemours, Wilmington, DE, USA. H_2_SO_4_ (98 wt%) and the other chemicals were provided from Sinopharm, Beijing, China. The components of ICRFB single-cell system were provided from Wuhan Chuxin Technology Co., Ltd., Wuhan, China.

### 2.2. Preparation of SPEEK

The PEEK 450PF powder was sulfonated with H_2_SO_4_ (98%) in a 60 °C water bath varied from 2 h to 6 h to prepare the SPEEK membranes with different DSs. With the glass rod moving, the solution was progressively poured into the ice-cold distilled water. Then the fibrous precipitate was washed to pH ≈ 7 with lots of distilled water and dried in the 80 °C vacuum oven for 12 h [28].

### 2.3. Preparation of SPEEK Membrane

**Figure 2:**
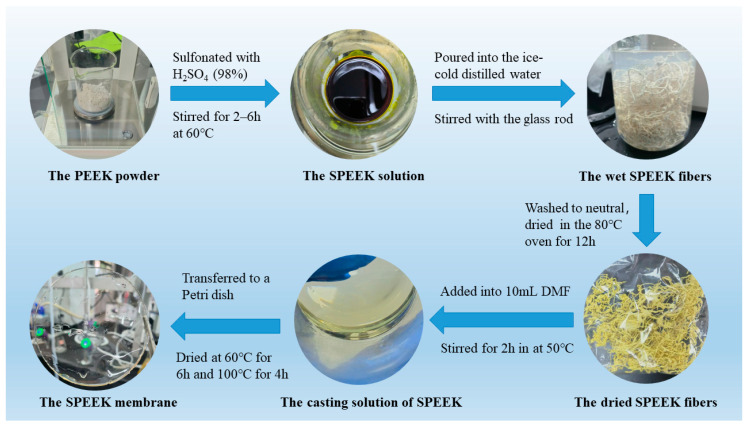
The preparation processes of SPEEK fibers and SPEEK membrane.

In total, 0.6 g SPEEK was added into 10 mL N, N′-dimethylformamide (DMF), and the solution was magnetically stirred for 2 h in a 50 °C water bath until the SPEEK was dissolved completely. Then the solution was transferred to a Petri dish and dried at 60 °C for 6 h and 100 °C for 4 h, respectively, in a vacuum oven. After the Petri dish was cooled to room temperature, it was further immerged in distilled water to separate the obtained sample. In order to remove the impurity and achieve the best property, the sample was immerged in 1 M sulfuric acid solution for 24 h. Finally, it was rinsed with distilled water till the pH reached neutrality and deposited in distilled water. Figure 2 shows the preparation processes of SPEEK polymer/fibers and SPEEK membrane.

### 2.4. Characterization

#### 2.4.1. Morphology and Fourier Transform Infrared (FTIR) Spectra

The cross-section morphology and energy dispersive X-ray spectroscopy (EDX) element mapping image was captured by scanning electron microscopy (SEM, JSM-7610F, JEOL, Tokyo, Japan). The FTIR was obtained using an FTIR spectrometer (Nicolet 6700, PerkinElmer, Shanghai, China) between 4000 and 450 cm^−1^ with 50 scans and 4 cm^−1^ resolution.

#### 2.4.2. Ion Exchange Capacity (IEC) and DS

To measure the IEC, the membrane was cut to pieces and immerged in a 25 mL saturated NaCl solution. After 12 h, the membrane was taken out and put into a 25 mL new saturated NaCl solution for 12 h. The whole soaking period is with magnetic stirring. Both of the solutions were mixed together, and 0.01 M sodium hydroxide solution was employed to titrate the mixed solution. The IEC is calculated by Equation (1) [29]:

(1) $$\mathrm{IEC}=\frac{V_{\mathrm{NaOH}}\times C_{\mathrm{NaOH}}}{W_{\mathrm{dry}}}$$

where W_dry_ is the weight of the dried membrane, V_NaOH_ and C_NaOH_ are the consumed volume and concentration of sodium hydroxide solution.

DS reflects the average number of -SO_3_ in each SPEEK module, and it is always calculated by Equation (2) [30]:

(2) $$\mathrm{DS}\;(\%)=\frac{\mathrm{IEC}\times {\mathrm{MW}}_{\mathrm{PEEK}}}{\mathrm{IEC}\times ({\mathrm{MW}}_{\mathrm{PEEK}}-{\mathrm{MW}}_{\mathrm{SPEEK}})+1000}\times 100\%$$

where MW_PEEK_ and MW_SPEEK_ are the molecular weights of the unsulfated and sulfonated monomers, IEC stands for the ion exchange capacity.

#### 2.4.3. Water Uptake (WU) and Swelling Ratio (SR)

The membrane was put into the vacuum oven (DZ–F6050BZ, Shanghai Yixin Scientific Instrument Co.Ltd., Shanghai, China) at 90 °C for 12 h, its weight and length were measured rapidly after the drying period. For wet membrane, it was immerged in the distilled water at 65 °C for 12 h, and quickly removed the moisture to measure. The WU and SR are obtained by Equations (3) and (4) [31]:

(3) $$\mathrm{WU}(\%)=\frac{W_{\mathrm{wet}}-W_{\mathrm{dry}}}{W_{\mathrm{dry}}}\times 100\%$$

(4) $$\mathrm{SR}\;(\%)=\frac{L_{\mathrm{wet}}-L_{\mathrm{dry}}}{L_{\mathrm{dry}}}\times 100\%$$

where W_dry_ and W_wet_ are the weights of dry and wet membranes, L_dry_ and L_wet_ are lengths of dry and wet membranes.

#### 2.4.4. Proton Conductivity, Cr^3+^/Fe^2+^ Permeability, Ion Selectivity, and Mechanical Properties

The sample was cut to a specific size (2 cm × 2 cm) and placed in the distilled water at 65 °C for 24 h, then we fixed it to a fixture which has dual platinum electrodes, and the fixture was immerged in the distilled water at 65 °C. The resistance was obtained by electrochemical impedance spectroscopy (EIS) through an electrochemical station (PGSTAT302N, Metrohm, Beijing, China). The conductivity is calculated by Equation (5) [32,33]:

(5) $$\sigma =\frac{a}{R\times l\times w}$$

where a is the distance of dual platinum electrodes, R, l, and w, are the resistance, thickness, and width of the membrane in the distilled water at 65 °C, respectively.

To take the permeability test, the membranes were sandwiched in the middle of an H-type electrolytic tank, which was filled with the solution of 25 mL 1 M CrCl_3_ + 3 M HCl on the left, and filled with 25 mL 1 M AlCl_3_ + 3 M HCl on the right (or 1 M FeCl_2_ + 3 M HCl on the left, 25 mL 1 M MnCl_2_ + 3 M HCl on the right). AlCl_3_ and MnCl_2_ are utilized to equalize the ionic strengths of the bilateral solutions and to minimize the osmotic pressure effect [22]. Both sides were stirred by the magnetic stirrer at 65 °C. At set intervals, 3 mL solution was taken to measure the concentration with the UV-vis spectrometer (SPECORD S600, Analytik Jena AG, Jena, Germany) and poured it back into the tank after measuring. The Cr^3+^/Fe^2+^ permeability is calculated by Equation (6) [34]:

(6) $$V_{R}\frac{d_{c_{R}}(t)}{\mathrm{dt}}=A\frac{P}{l}[c_{L}-c_{R}(t)]$$

where P is Cr^3+^/Fe^2+^ permeability, V_R_ is the volume of AlCl_3_/MnCl_2_ solution, C_L_ and C_R_(t) are Cr^3+^/Fe^2+^ concentration in CrCl_3_/FeCl_2_ and AlCl_3_/MnCl_2_ solutions. L and A are thickness and area of the membrane. 

The ion selectivity is determined by the proton conductivity and Cr^3+^/Fe^2+^ permeability, and it is calculated by Equation (7) [35]:

(7) $$S=\frac{\sigma }{P}$$

where P is Cr^3+^/Fe^2+^ permeability, σ is the proton conductivity.

The membranes were dried in the 90 °C vacuum oven for 12 h and cut into a rectangle shape with the size of 50 mm × 10 mm. The prepared membranes were tested by the DR-509A electromechanical universal testing machine (Dongri Instrument Co., Ltd., Dongguan, China) to obtain the mechanical properties, and the tensile speed was set as 50 mm min^−1^.

#### 2.4.5. Chemical Stability Test

The sample was immerged in a 50 mL cathode/anolyte at 65 °C, and the electrolyte was composed of 1 M FeCl_2_ + 1 M CrCl_3_ + 3 M HCl. The mass loss and length loss of the membrane are calculated by Equations (8) and (9) [22], respectively:

(8) $$\mathrm{Weight}\;\mathrm{loss}\;(\%)=\frac{w_{1}-w_{0}}{w_{0}}\times 100\%$$

(9) $$\mathrm{Length}\;\mathrm{loss}\;(\%)=\frac{L_{1}-L_{0}}{L_{0}}\times 100\%$$

where w_0_ and w_1_ are the weights of the membrane before and after immerging in the cathode/anolyte. L_0_ and L_1_ are the lengths of the membrane before and after immerging in the cathode/anolyte.

### 2.5. CV Tests of the Iron-Chromium Redox Species

The characteristics of the iron-chromium redox species in the electrolyte are determined by cyclic voltammetry (CV) through an electrochemical workstation CHI760E of Shanghai CH Instrument Co., Ltd. (Shanghai, China). The experiment was carried out in the standard three-electrode system at ambient temperature [36]. The reference and counter electrodes were calomel electrode and platinum sheet electrode, respectively, and the working electrode was the graphite felt, which size was 1.5 cm × 2.0 cm. The scan window of the CV curve is limited to 0.0~0.8 V and −0.8~0.0 V.

### 2.6. ICRFB Single-Cell Performance

**Figure 3:**
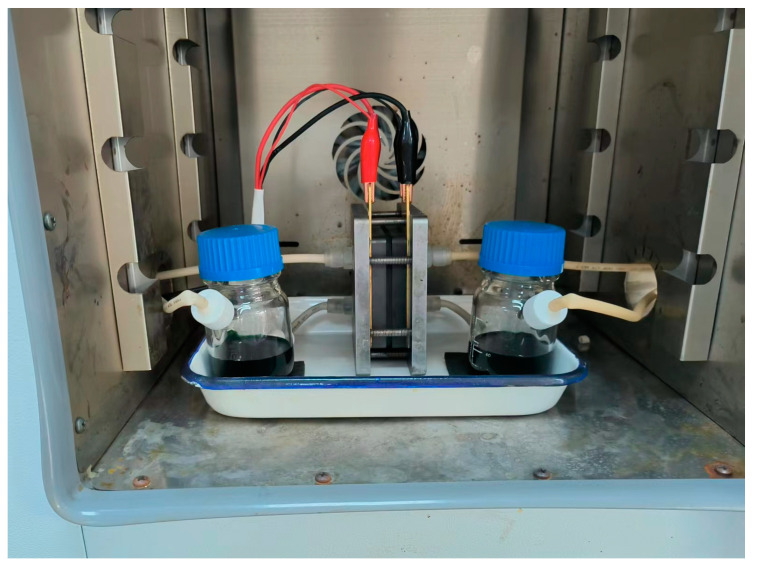
Photograph of the ICRFB single-cell.

The ICRFB was built up by sandwiching the membrane with a 5 cm × 5 cm active area between double 4.5 mm thick graphite felts. Two copper foils and graphite bipolar plates were used as the current collectors. In total, 40 mL electrolyte consisting of 1 M FeCl_2_ + 1 M CrCl_3_ + 3 M HCl served as the cathode/anolyte [37], and they were transported by the peristaltic pump (DIPump550, Kamoer, Shanghai, China), the flow velocity was set at 100 rpm (115 mL/min). Figure 3 shows the photograph of the ICRFB single-cell.

The stack resistance of each single-cell reflects the electrochemical performance of the integral battery system, which was obtained by the RC3563 battery tester (Leadop Co., Ltd., Shanghai, China) The ICRFB single-cell with various membranes ran without power for a period of time, then recorded the stabilized value as the stack resistance. 

The single-cell was operated by a Land-CT3002K (Wuhan Land Co., Ltd., Wuhan, China). The current density of the charge–discharge cycling tests ranged from 40 to 100 mA cm^−2^ or from 40 to 120 mA cm^−2^, and set 20 mA cm^−2^ as the interval. The lower limit of discharge voltage was 0.8 V and the upper limit of charge voltage was 1.2 V. The CE, voltage efficiency (VE) and EE are obtained by Equations (10)–(12) [38]:

(10) $$\mathrm{CE}=\frac{\int I_{d}dt}{\int I_{c}dt}\times \;100\%$$

(11) $$\mathrm{VE}=\frac{\int V_{d}I_{d}dt}{\int V_{c}I_{c}dt}\times 100\;\%$$

(12) EE=CE×VE

where I_d_ and I_c_ are the discharge current and charge current. V_d_ and V_c_ are the discharge voltage and charge voltage.

## 3. Results and Discussion

### 3.1. Characterization

#### 3.1.1. Morphology and FTIR

**Figure 4:**
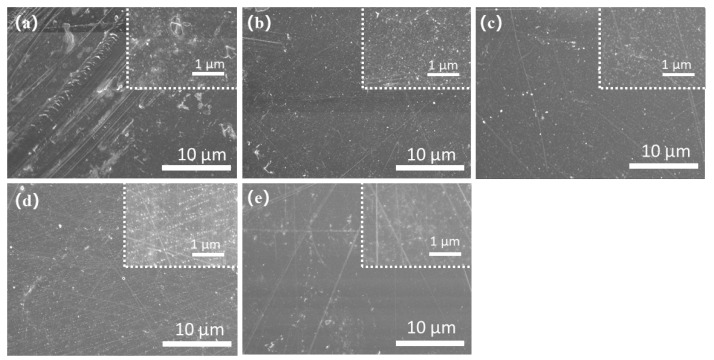
SEM images of the surface of the SPEEK membranes: (**a**) SPEEK 43; (**b**) SPEEK 47; (**c**) SPEEK 52; (**d**) SPEEK 57; (**e**) SPEEK 62.

**Figure 5:**
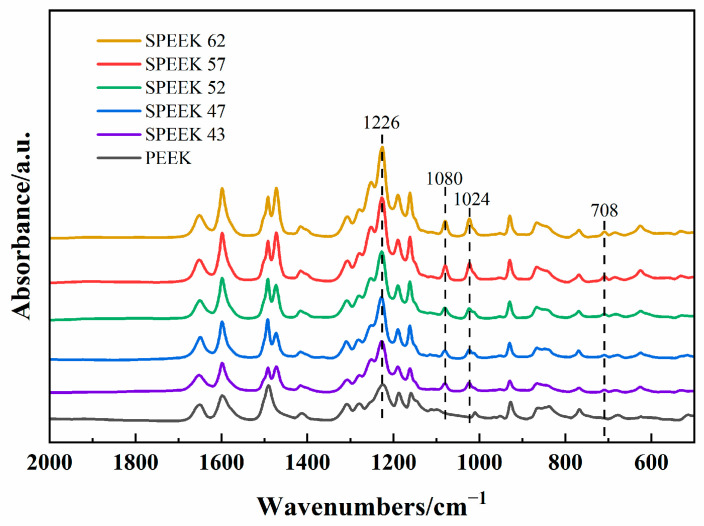
FTIR spectra.

The morphologies of five DSs SPEEK membranes can be seen in Figure 4a–e. According to Figure 4a, SPEEK 43 presents the coarsest morphology with the other membrane presenting a relatively smooth surface as Figure 4b–e. This phenomenon is because when the DS is too low, the solution of SPEEK in DMF or the other organic solvents will no longer be clear and appears milky [39] as observed during the process of the preparation. The FTIR results of five DSs SPEEK and the pure PEEK powder are shown in Figure 5. The addition of -SO_3_ groups during the sulfonation process makes SPEEK exhibit four new absorption peaks, including S-O stretching (708 cm^−1^), S=O stretching (1024 cm^−1^), symmetric stretching of O=S=O (1080 cm^−1^), and asymmetric of O=S=O (1226 cm^−1^) [24,40]. These characterizations reflect that the -SO_3_ groups are added to the SPEEK membranes successfully. Moreover, the increase in these peak intensities is corresponds to an increase in DS, demonstrating the concordance of membrane and properties.

#### 3.1.2. Physicochemical Properties, Cr^3+^/Fe^2+^ Permeability, and Ion Selectivity

**Figure 6:**
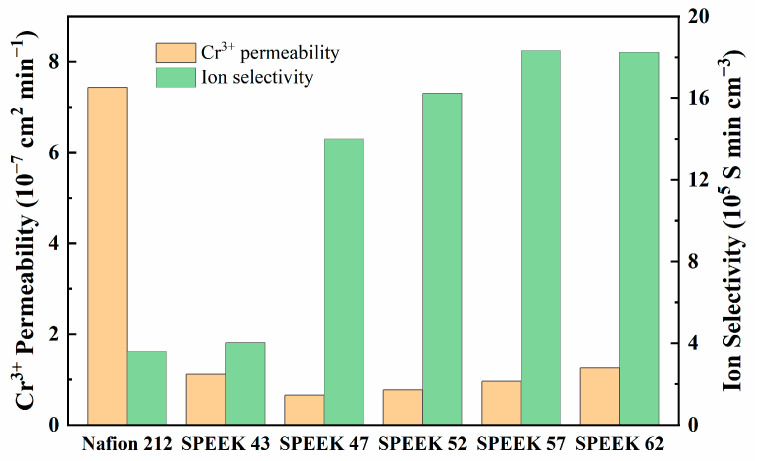
Cr^3+^ permeability and ion selectivity.

Table 1 exhibits the physicochemical properties of five DSs SPEEK membranes and Nafion 212. As DS increases, IEC, proton conductivity, WU, and SR of the membranes are higher as well, especially based on the results of SPEEK 52 and SPEEK 57. The results demonstrate that the physicochemical variation in the SPEEK membranes is nonlinear. As Figure 6 shows, all the SPEEK membranes present a significantly lower Cr^3+^ permeability than the Nafion 212; this is because the steric hindrance which is produced by the aromatic ring structures of the SPEEK membranes reduces the Cr^3+^ permeability [41]. However, the Cr^3+^ permeability of SPEEK 43 is higher than that of SPEEK 57, which may be due to the defective morphology that was observed in Figure 4a. On the one hand, the higher DS brings higher WU so as to reach better proton conductivity, but the worse stability and higher Cr^3+^ permeability are also inevitable due to the higher SR [42]. On the other hand, too low DS not only reduces the proton conductivity but also elevates the Cr^3+^ permeability, leading to the worst ion selectivity afterward. Therefore, the DS of SPEEK membranes should be moderate to achieve the best combination property. The Fe^2+^ permeability behavior of the various membranes is attached in Figure S1, and it is obviously lower than that of the Cr^3+^. However, the permeability and ion selectivity for the membranes exhibit similar tendencies compared with the results of Cr^3+^, which further prove the different ion-selective ability of the membranes.

**Figure 7:**
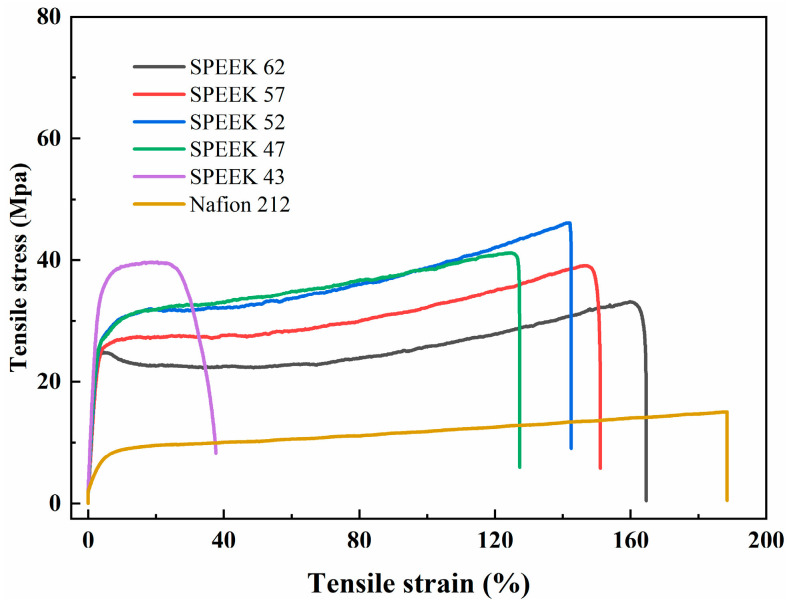
Stress-strain curves.

The mechanical properties are crucial for the proton exchange membranes, and they are shown in Table 1 and Figure 7. It could be found that all the SPEEK membranes present a higher tensile strength and Young’s modulus than the Nafion 212, which was owing to their higher rigidity resulting from the rigid aromatic ring structure [24]. With the increase in the DS, the tensile strength of the SPEEK membranes exhibits a tendency to climb up and then decline while the percentage elongation progressively grows. Considering the rigidity and ductility of the SPEEK membranes comprehensively, the mechanical properties of SPEEK 57 are the most balanced.

#### 3.1.3. Evaluation of Chemical Stability

**Figure 8:**
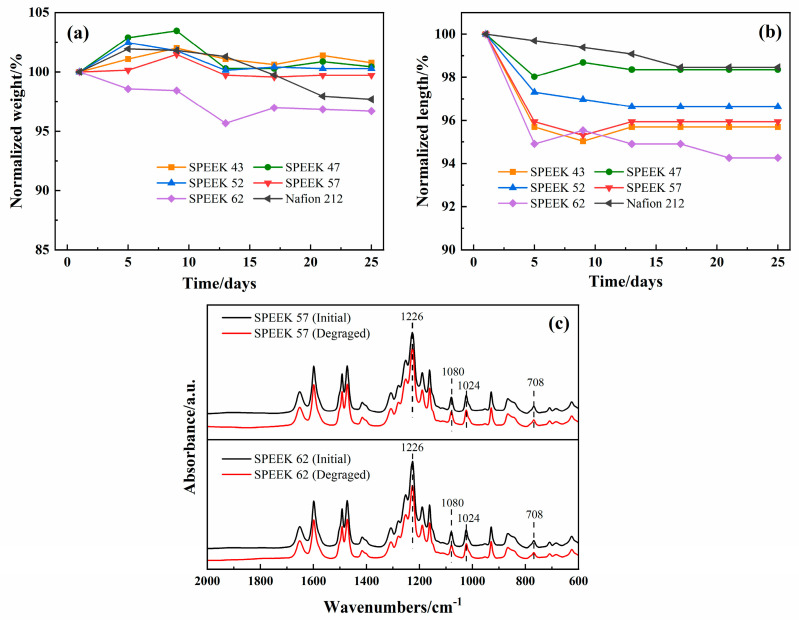
Relative variations of SPEEK membranes with various DSs and Nafion 212 in: (**a**) weight and (**b**) length; (**c**) FTIR spectra of SPEEK 57 and SPEEK 62 before and after the chemical stability test.

The past works confirm that 65 °C is the best operating temperature for ICRFB [43], and this condition has increased the requirement for the chemical stability of the membranes. All the various DSs SPEEK and Nafion 212 membranes were immersed in the electrolyte at 65 °C for 25 days. The outcomes are demonstrated in Figure 8a,b, and it is intuitive to see both length and weight tend to be stable for all the membranes after half a month. For the normalized weight, SPEEK 62 decreases the most, Nafion 212 decreases the second, and the decrease in SPEEK 57 is close to SPEEK 52. For the normalized length, SPEEK 62 still presents the most decrease, while Nafion 212 presents the least. These results indicate that SPEEK 62 has the worst chemical stability, and the other SPEEK membranes have advantages in terms of chemical stability for weight compared with the Nafion 212.

**Figure 9:**
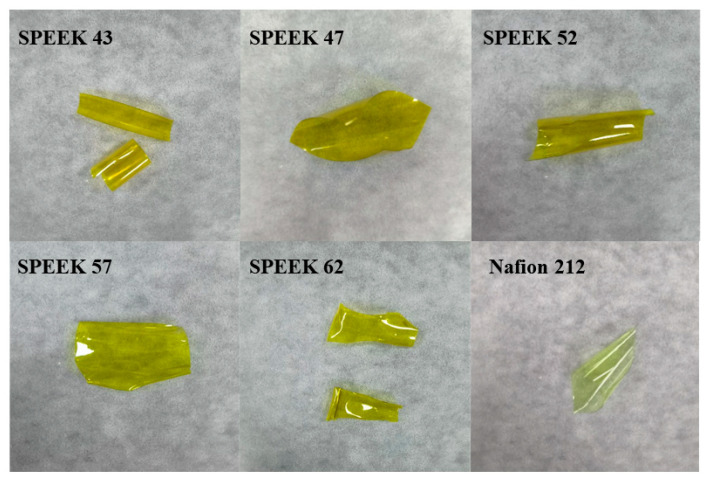
Photographs of SPEEK membranes with various DSs and Nafion 212 after the chemical stability test.

In order to investigate the chemical stability of SPEEK membranes further, the FTIR tests of SPEEK 62 and SPEEK 57 were conducted, which exhibit the most noticeable variations of weight and length at the above tests. As shown in Figure 8c, SPEEK 62 shows a slight change in the -SO_3_H characteristic peaks, while SPEEK 57 shows no noticeable change, indicating that the sulfonic acid groups of the SPEEK membranes remain relatively stable during the long-term immersion of iron-chromium electrolyte at 65 °C [22,44]. The photographs of SPEEK membranes with various DSs and Nafion 212 after the chemical stability test are shown in Figure 9. Obviously, the SPEEK 62 presents the most flawed shape than the other membranes, and this is consistent with the results of Figure 8.

### 3.2. CV Tests of the Iron-Chromium Redox Species

**Figure 10:**
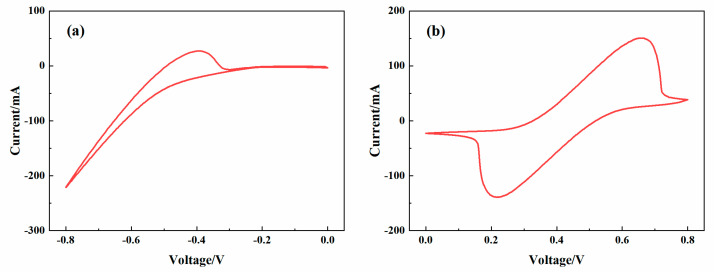
CV curves of the electrolyte with 1 M FeCl_2_ + 1 M CrCl_3_ + 3 M HCl: (**a**) Cr^3+^/Cr^2+^ redox reaction at the scanning of 1 mV/s; (**b**) Fe^2+^/Fe^3+^ redox reaction at the scanning of 3 mV/s.

The redox reactions of Cr^3+^/Cr^2+^ and Fe^2+^/Fe^3+^ are shown in the Figure 10a,b. The peak current of Cr^3+^/Cr^2+^ is I_pa_ = 51.7 mA, the peak currents of Fe^2+^/Fe^3+^ are I_pa_ = 150.3 mA and I_pc_= −138.7 mA. These results show that the prepared electrolyte has great electrochemical activity to be used in the ICRFB single-cell performance tests [37].

### 3.3. ICRFB Single-Cell Performance

At first, SPEEK 62, SPEEK 57, and SPEEK 52 membranes were chosen to conduct the ICRFB single-cell tests because they have the top three ion selectivity. According to Figure S2, SPEEK 62 demonstrates the best VE and EE in the different current densities while SPEEK 57 exhibits the best CE. It is worth noting that despite SPEEK 52 having the lowest Cr^3+^ permeability, as shown in Figure 6, it still possesses the worst CE compared with the other two membranes. This is because the CE is also influenced by the resistance of the membranes, when the resistance is too high, there will be more side reactions in the electrode, and the CE will be worse [45,46].

Considering the chemical stability, physicochemical properties, and the actual performance of the SPEEK membranes synthetically, new and thinner membranes of SPEEK 52 (thickness15 μm, SPEEK 52-15 for short) and SPEEK 57 (thickness 25 μm, SPEEK 57-25 for short) were prepared to decrease the resistance [47]. The physicochemical characteristics, such as IEC and WU, are not influenced by the thickness, all the values are the result of the homogenization of thickness, size, and weight, thus similar tests were not carried out for the thinner membranes again. The stack resistances of all the single-cells assembled with SPEEK membranes and Nafion 212 membranes are shown in Table 2; it is obvious that the stack resistance of the cells with SPEEK membranes does not vary linearly with the DS. It improves significantly when the DS declines from 57% to 52%, and the single-cell with SPEEK 43 membrane exhibits the highest stack resistance due to its coarse structure. Additionally, the stack resistance of single-cells with SPEEK 57-25 and SPEEK 52-15 membranes declines obviously compared with the original SPEEK 57 and SPEEK 52, which suggests that the reduction in thickness is worked. The next ICRFB single-cell tests will reveal their actual performance during the operating state.

**Figure 11:**
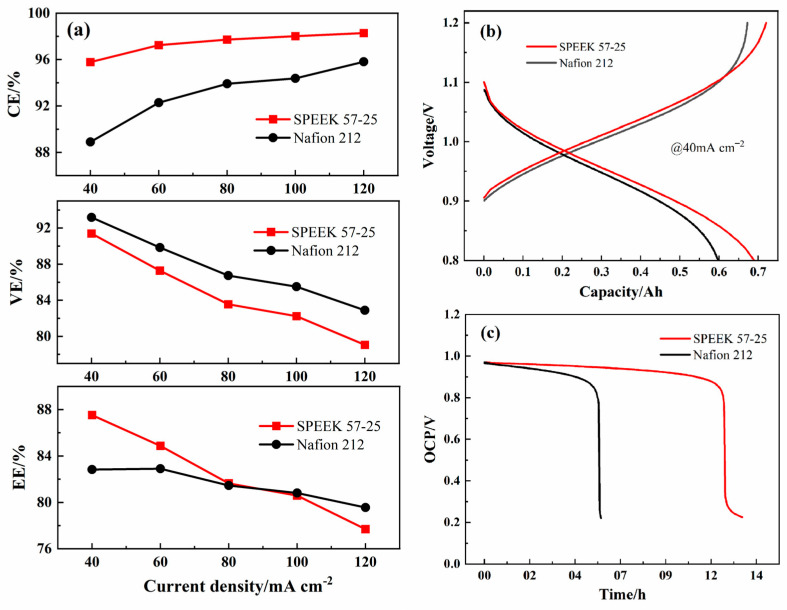
(**a**) The performance of ICRFB single-cells assembled with Nafion 212 and SPEEK 57-25 membranes under various current densities; (**b**) single-cycle charge–discharge curves of ICRFB at 40 mA cm^−2^; (**c**) self-discharge curves of ICRFB by OCP tests.

The ICRFB single-cell performance of SPEEK 52-15 is shown in Figure S3; though the ultrathin thickness elevates the VE significantly, the CE also decreases dramatically due to the crossover process taking place with more ease, which results in the EE still being bad (under 80% at all the current densities). On the other hand, the SPEEK 57-25 presents an excellent EE and VE, while the CE has a small reduction, as shown in Figure 11a. Compared with Nafion 212, SPEEK 57-25 membrane presents a better CE during all the current densities, a better EE during from 40 mA cm^−2^ to 80 mA cm^−2^, and the gap is increasing with the decline of the current density. When it exceeds 100 mA cm^−2^, the EE of Nafion 212 had reversed, which means that the resistance of the SPEEK 57-25 should be further decreased by the conductive materials [48,49,50,51,52] in future works to ensure it performs better at higher current densities.

The charge–discharge capacity curves of the two membranes under 40 mA cm^−2^ have been compared as well, the cycling capacity of the SPEEK 57-25 is greater than that of the Nafion 212 as shown in Figure 11b. The charge voltage of Nafion 212 is lower at first but higher than that of SPEEK 57-25 at last. This is because the Nafion 212 has a lower ion selectivity and a lower EE, which leads to the ICRFB assembled with Nafion 212 has a higher internal resistance at the late reaction period [53]. Additionally, the lower metal ions permeability makes SPEEK 57-25 own a higher discharge voltage during all the periods. The open circuit potential (OCP) curves of ICRFB assembled with these two membranes are shown in Figure 11c, and these two ICRFBs were firstly charged to a state of charge (SOC) ca. 50% at 80 mA cm^−2^. After 6 h, the ICRFB assembled with Nafion 212 first dropped sharply to 0.2 V, while the ICRFB assembled with SPEEK 57-25 dropped sharply to 0.2 V at a time more than twice as long compared to that of the Nafion 212. This longer discharge time indicates that the metal ions permeability of SPEEK 57-25 is still far lower than the Nafion 212 even though it has an ultrathin thickness [54,55], and it is consistent with the results of Figure 6, as shown above.

**Figure 12:**
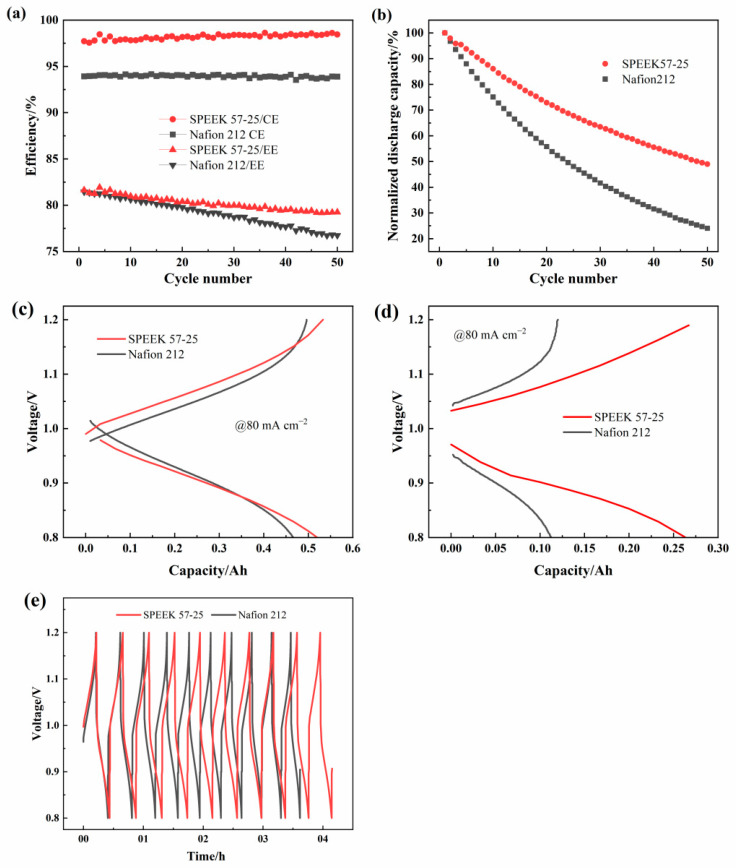
Cycling performance of ICRFBs with Nafion 212 and SPEEK 57-25 under 80 mA cm^−2^: (**a**) CE and EE; (**b**) normalized discharge capacity variations; (**c**) the first cycle charge–discharge curves; (**d**) the 50th cycle charge–discharge curves; (**e**) voltage-time curves of the first 10 cycles.

To explore the oxidation stability, 50 charge–discharge cycles of the ICRFBs built up with these two membranes under 80 mA cm^−2^ were carried out. As shown in Figure 12a, both of the membranes present relatively stable CE during the 50 cycles. However, the EE of these two membranes appears marked decline tendency, especially for the Nafion 212. It started out a little lower than SPEEK 57-25 but ended up being significantly lower after 50 cycles. The capacity retention of charge–discharge cycling is influenced by the permeation of active ions (Fe and Cr ions) [56] and the side reactions (e.g., air oxidation of Fe and Cr ions [57] and hydrogen evolution [58]). According to Figure 12b, SPEEK 57-25 demonstrates a lower discharge capacity decay (50%) than the Nafion 212 (76%), which further proves the lower metal ions permeability of SPEEK 57-25 [59]. Figure 12c,d shows the charge–discharge curves for the 1st and 50th cycles to demonstrate the membrane stability in more detail. The first charge–discharge curve is close to the condition of the 40 mA cm^−2^ current density as Figure 11b, while the 50th charge–discharge curve of SPEEK 57-25 exhibits a significantly lower charge voltage and higher discharge voltage than that of the Nafion 212. These results indicate that with the increase in the cycle numbers, the charge–discharge capacity of the Nafion 212 will be exceedingly low, which proves the extremely strong metal ions permeability and poor ion selectivity of Nafion 212 compared with the SPEEK 57-25. In addition, the voltage-time curves of the first 10 cycles are reported in Figure 12e. It is clear that SPEEK 57-25 has a longer operating time following the variation in voltage at the same cycle number, which brings a longer charge–discharge time and a great charge–discharge capacity retention.

**Figure 13:**
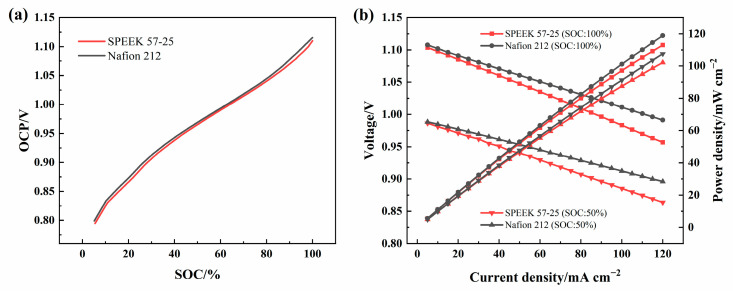
(**a**) OCP-SOC curves of SPEEK 57-25 and Nafion 212 under 80 mA cm^−2^; (**b**) the polarization curves of the cells under 100% SOC and 50% SOC with SPEEK 57-25 and Nafion 212.

Figure 13a shows the OCP-SOC curves of SPEEK 57-25 and Nafion 212, the ICRFB single-cells were gradually charged to a specific capacity, and rested for 30 s at each step to record the OCP precisely. The result shows that the two membranes have a similar OCP at the same SOC, and Nafion 212 is slightly higher. The polarization tests of the two membranes were operated as follows: the single-cells were charged to SOC = 100% or SOC = 50% at 80 mA cm^−2^, and they were discharged for 30 s to acquire a median voltage, the interval of the discharge current density was set as 5 mA cm^−2^. To ensure the SOC was unchanged, the cells were charged to 1.2 V at 80 mA cm^−2^ after each period of discharge. The experiments above show that the performance of the single-cells with the two membranes will both be worse when the current density is higher than 120 mA cm^−2^, and the electrodes used in this research may not be able to withstand the higher currents, so 120 mA cm^−2^ was set as the upper limit of the polarization test. 

As Figure 13b shows, with the current density increasing, the voltages of the single-cells gradually decrease after the discharge steps. This is because, during the same time, the cell with a higher current density releases more electricity, making the voltage lower. However, the voltage is also affected by the ohmic polarization of the electrode, electrolyte, and membrane. When another condition is identical, the higher internal resistance of the membrane will cause greater transfer resistance (ohmic polarization) [60] of protons during the transmission process, resulting in voltage loss and decreasing the power density and EE of the single-cell. Taken together with the VE of Figure 11a, the decrease in the resistance of SPEEK 57-25 should be further considered. 

**Figure 14:**
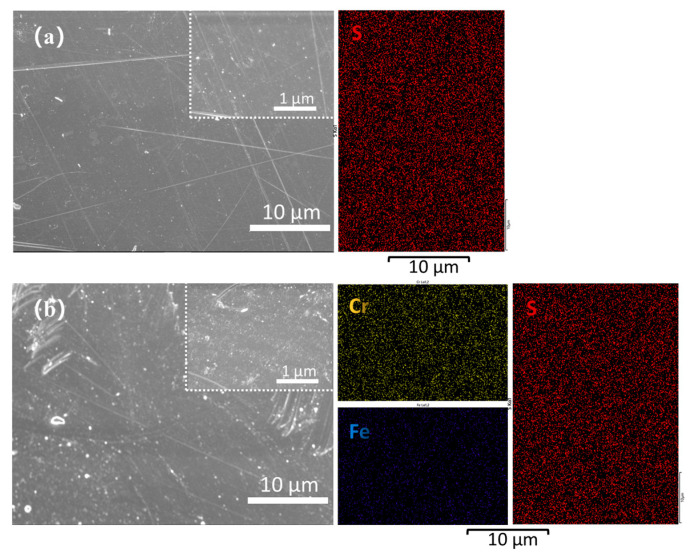
(**a**) SEM and EDX images of the surface of the initial SPEEK 57-25 membrane; (**b**) SEM and EDX images of the surface of the SPEEK 57-25 membrane after all the cycling tests.

To intuitively observe the structure of the SPEEK 57-25 before and after the single-cell tests, the EDX element and morphology images are shown in Figure 14a,b. The results indicate that after the impact of the strong oxidizing electrolyte at high temperatures for days, the tested membrane is not as smooth as the initial state, but its surface does not show obvious defects. In addition, the EDX images of the slightly changed element S and uniformly distributed elements of Fe and Cr ulteriorly prove the excellent stability of SPEEK 57-25.

## 4. Conclusions

In this study, the properties of SPEEK membranes with five various DSs were tested for the ICRFB applications. The experiment results show that a high DS may break the chemical stability, and a low DS influences the morphology so as to abate the ion selectivity of the membranes. Compared to the other SPEEK membranes, SPEEK 57 exhibits the most balanced physicochemical properties. The ICRFB single-cell assembled with the SPEEK 57-25 membrane presented an excellent cell performance (CE: 95.78–98.28%, EE: 77.69–87.53%) under 40–120 mA cm^−2^. In contrast with Nafion 212, the cheaper price, excellent physicochemical characteristics, longer self-discharge time, and lower capacity decay after 50 cycles make SPEEK 57-25 own the practical potential to be employed for large-scale ICRFB applications in the future. In the next work, it is necessary to add some conductive materials to further decrease the resistance of the membrane to obtain a more excellent performance at a high current density.

## Figures and Tables

**Table 1:** Physicochemical properties of Nafion 212 and five DSs SPEEK membranes.

Membrane	Thickness (Wet, 65 °C, μm)	IEC (mmol g^−1^)	DS (%)	Proton Conductivity (S cm^−1^)	WU% (65 °C)	SR% (65 °C)	Tensile Strength (Mpa)	Young’s Modulus (Mpa)	Percentage Elongation (%)
Nafion 212	55	0.99	-	0.267	12.2	10.8	15.1	144.4	187.8
SPEEK 43	55.5	1.33	42.9	0.045	23.4	4.9	39.7	1139.5	37.7
SPEEK 47	55	1.44	46.8	0.091	27.7	6.7	41.2	989.5	127.3
SPEEK 52	56.5	1.58	52.1	0.125	30.2	9.0	46.1	921.8	142.5
SPEEK 57	56	1.72	57.4	0.176	41.5	13.5	39.1	860.4	151.1
SPEEK 62	57	1.84	62.1	0.230	56.5	16.4	33.1	835.3	164.9

**Table 2:** Stack resistance of all the SPEEK membranes and Nafion 212 single-cells.

Membrane	Nafion 212	SPEEK 43	SPEEK 47	SPEEK 52	SPEEK 57	SPEEK 62	SPEEK 52-15	SPEEK 57-25
Stack resistance (mΩ)	22.89	137.94	96.08	78.05	55.87	44.95	32.96	31.13

## Data Availability

Data are contained within the article or Supplementary Materials.

## Supplementary Materials

The following supporting information can be downloaded at: https://www.mdpi.com/article/10.3390/membranes13100820/s1, Figure S1: Fe^2+^ permeability and ion selectivity. Figure S2: The performance of ICRFB single-cell with SPEEK 52, SPEEK 57, and SPEEK 62 membranes under different current densities; Figure S3: The performance of ICRFB single-cell with SPEEK 52-15 under different current densities.
