# Supplementary material for: A Comparative Study of Nafion 212 and Sulfonated Poly(Ether Ether Ketone) Membranes with Different Degrees of Sulfonation on the Performance of Iron-Chromium Redox Flow Battery
Source: Membranes (Basel). 2023 Sep 30;13(10):820. doi: 10.3390/membranes13100820 (PMC10608269; doi:10.3390/membranes13100820)
Supplement: Supplementary file 1 [file membranes-13-00820-s001.zip › membranes-2612014-supplementary.pdf]

membranes

Article

A comparative study of Nafion 212 and sulfonated poly(ether ether ketone) membranes with different degrees of sulfonation on the performance of iron-chromium redox flow battery

Enrui Bai[1], Haotian Zhu[1,2], Chuanyu Sun[3], Guanchen Liu[4], Xiaoyin Xie[2,*], Chongyang Xu[1,*], Sheng Wu[1,*]

1 Yantai Research Institute, Harbin Engineering University, Yantai Shandong 264003, China; S321517001@163.com(E.B.); zht18732497702@163.com(H.Z.)
2 School of Chemistry and Chemical Technology, Hubei Polytechnic University, Huangshi 435003, China;
3 School of Electrical Engineering and Automation, Harbin Institute of Technology, Harbin 150001, China; chuanyu.sun@hit.edu.cn
4 Hubei Xinye Energy-storage Co.,Ltd., Huangshi 435100, China; liuguanchen1123@163.com
* Correspondence: xyxie@hbpu.edu.cn(X.X.); xcy110@126.com(C.X.); wusheng@hrbeu.edu.cn(S.W.); Tel.: +86-15549716559(X.X.)

This word includes:

Figure S1, Figure S2 and Figure S3

Citation: Bai, E.; Zhu, H.; Sun, C.; Liu, G.; Xie, X.; Xu, C.; Wu, S. A comparative study of Nafion 212 and sulfonated poly(ether ether ketone) membranes with different degrees of sulfonation on the performance of iron-chromium redox flow battery. *Membranes* **2023**, *13*, 820. https://doi.org/10.3390/membranes13100820

Academic Editor: Vladislav A. Sadykov

Received: 30 August 2023
Revised: 24 September 2023
Accepted: 28 September 2023
Published: 30 September 2023

Copyright: © 2023 by the authors. Licensee MDPI, Basel, Switzerland. This article is an open access article distributed under the terms and conditions of the Creative Commons Attribution (CC BY) license (https://creativecommons.org/licenses/by/4.0/).

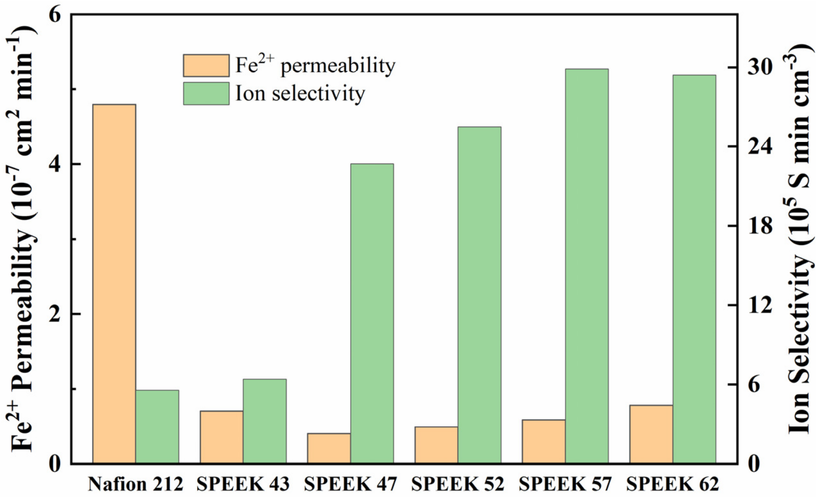

Figure S1. Fe^{2+} permeability and ion selectivity.

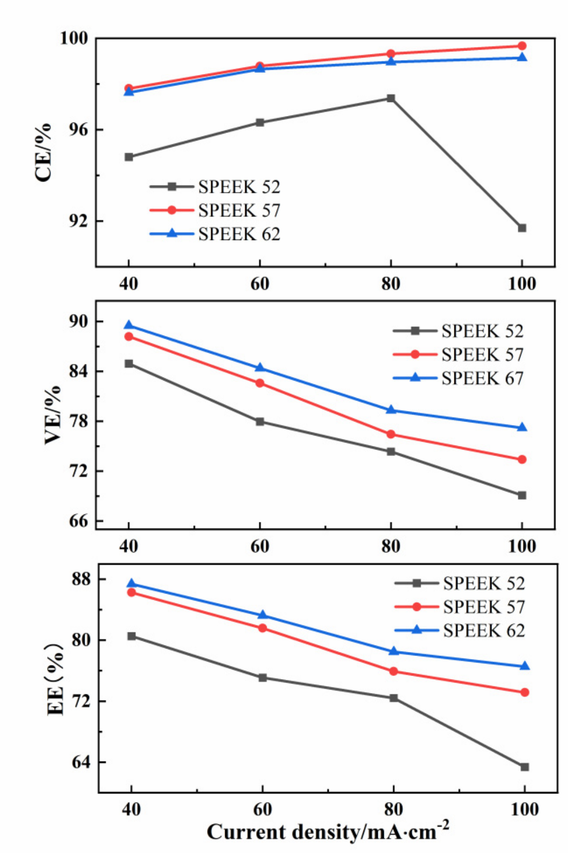

Figure S2. The performance of ICRFB single-cell with SPEEK 52, SPEEK 57 and SPEEK 62 membranes under different current densities.

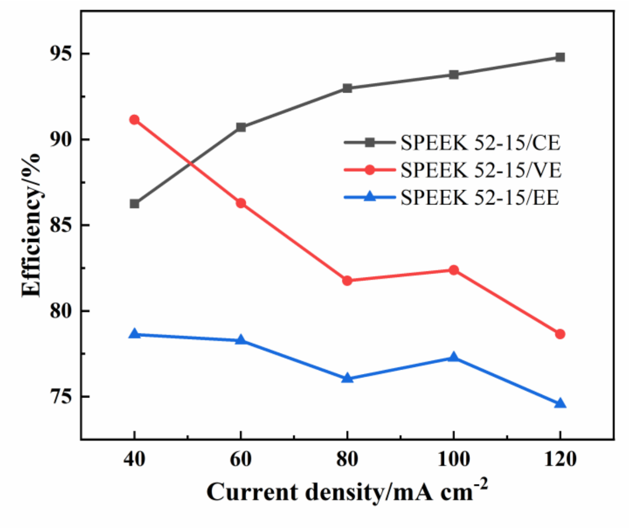

Figure S3. The performance of ICRFB single-cell with SPEEK 52-15 under different current densities.
